# Dexmedetomidine Alleviated Endoplasmic Reticulum Stress via Inducing ER-phagy in the Spinal Cord of Neuropathic Pain Model

**DOI:** 10.3389/fnins.2020.00090

**Published:** 2020-02-28

**Authors:** Yongda Liu, Shuang Wang, Zhibin Wang, Mengmeng Ding, Xingyue Li, Jiao Guo, Guang Han, Ping Zhao

**Affiliations:** ^1^Department of Anesthesiology, Shengjing Hospital of China Medical University, Shenyang, China; ^2^Department of Pain Management, Shengjing Hospital of China Medical University, Shenyang, China; ^3^Department of Anesthesiology, First Affiliated Hospital of China Medical University, Shenyang, China

**Keywords:** ER stress, autophagy, ER-phagy, neuropathic pain, spinal cord, dexmedetomidine

## Abstract

Studies demonstrated that spinal autophagy was impaired in spinal nerve ligation (SNL) rats. However, the relationship of endoplasmic reticulum (ER) stress and ER-phagy and whether dexmedetomidine (DEX) modulates ER-phagy remain unclear. In this study, male Sprague–Dawley (SD) rats and the SNL animal model were used. 4-Phenylbutyric acid (4-PBA), tunicamycin (TM), rapamycin (RAP), and 3-methyladenine (3-MA) were intrathecally administered, respectively to demonstrate the relationship of ER stress and ER-phagy. Dexmedetomidine (30 μg/kg) was administered as treatment. Mechanical withdrawal threshold (MWT) and thermal withdrawal latency (TWL) tests were performed to evaluate nociceptive hypersensitivity. Protein expressions were examined by Western blot, and the location of glucose-regulated protein 78 (Grp78) was examined by immunofluorescence staining. SNL induced ER stress and ER-phagy impairment. ER stress was altered in rostral ventromedial medulla (RVM); 4-phenylbutyric acid induced analgesic effect via inhibiting ER stress and unfolded protein response (UPR) pathways to induce ER-phagy; tunicamycin led to worsening pain through enhancing ER stress and UPR pathways to further impair ER-phagy. Rapamycin provided analgesic effect through enhancing ER-phagy to relieve SNL-induced ER stress and UPR pathway activation; 3-methyladenine deteriorated pain via further impairing ER-phagy to aggravate ER stress. Dexmedetomidine provided analgesic effect through elevating ER-phagy. In conclusion, ER stress led to ER-phagy impairment in the spinal cord of SNL rats and participated in the nociceptive descending system. ER-phagy impairment was both a trigger and an effector of ER stress via UPR pathways in SNL rats. Dexmedetomidine targeted ER-phagy to provide analgesic effect.

## Introduction

Cellular stress, free radical exposure, calcium depletion, and unfolded or misfolded protein accumulation in endoplasmic reticulum (ER) lumen lead to ER stress (Sisinni et al., 2019). Recent studies demonstrated that ER stress might be involved in the induction and maintenance of neuropathic pain (Ge et al., 2018). The unfolded protein response (UPR) is a response process that plays a critical role in restoring homeostasis following ER stress (Geronimo-Olvera and Massieu, 2019). ER stress leads to recruit glucose-regulated protein 78 (Grp78, a chaperone that is induced by the UPR and is also recognized as a regulator of UPR activation) and release UPR sensors: protein kinase RNA-like ER kinase (PERK), inositol-requiring enzyme 1 (IRE1), and ATF6. In this study, we examined PERK/activating transcription factor (ATF) 4, IRE-1/c-Jun N-terminal kinase (JNK), and ATF6 pathways to reveal the possible mechanism underlying UPR and autophagy (Sisinni et al., 2019).

Autophagy (macroautophagy) controls homeostasis in various biological and pathological conditions, which could be a non-selective mechanism that degrades general cytosol, proteins, and organelles by lysosomes. Studies suggested that autophagy might provide a protective mechanism in neuropathic pain animal models and clinical trials (Liu X. et al., 2019). The expression of autophagic markers microtubule-associated protein II light chain 3 (LC3) and p62 was examined to evaluate the autophagy level in spinal cord. LC3 is the most widely investigated marker in understanding autophagy, which is capable of evaluating the level of autophagysomes (Forrester et al., 2019). p62/Sequestosome 1 (SQSTM1) can bind to LC3, incorporate into autophagosomes directly, and be degraded by autophagy process (Lim et al., 2014).

Selective autophagy is mediated by autophagy receptors that simultaneously incorporate the specified substrates into autophagosomes (Wilkinson, 2019b). ER is selectively recognized and degraded by autophagy, in this case, called ER-phagy. In mammals, the FAM134B receptor is the first one to be used to evaluate ER-phagy level (Grumati et al., 2018). Autophagy might be a pro-survival or a pro-death process in different conditions. In this case, we evaluated apoptosis level by examining cleaved caspase-3 (Zhou et al., 2016; Liu et al., 2018).

Our previous studies demonstrated that spinal autophagy was impaired in SNL rats and enhanced autophagy might provide a protective effect in SNL-induced neuropathic pain (Liu et al., 2017; Liu Y.D. et al., 2019). However, the relationship between ER stress and ER-phagy in neuropathic pain circuits remains unexplained in this animal model. To determine the relationship of ER stress and ER-phagy in nociceptive modulatory system, we evaluated ER stress and ER-phagy in the spinal cord of SNL-induced neuropathic pain. In addition, we examined the confocal immunofluorescence of Grp78 (ER stress marker) and NeuN (neuron marker) in rostral ventromedial medulla (RVM) to investigate whether ER stress was altered in nociceptive descending modulation system. Dexmedetomidine is a widely used drug in the operation room for general anesthesia, but its analgesic effect mechanism for neuropathic pain remains unknown, Moreover, in this study, we demonstrated that dexmedetomidine targeted ER-phagy to provide analgesic effect, suggesting a novel insight and mechanism of dexmedetomidine. Above, ER-phagy in neuropathic pain might be a promising target for novel therapy.

## Materials and Methods

### Animals

**FIGURE 1:**
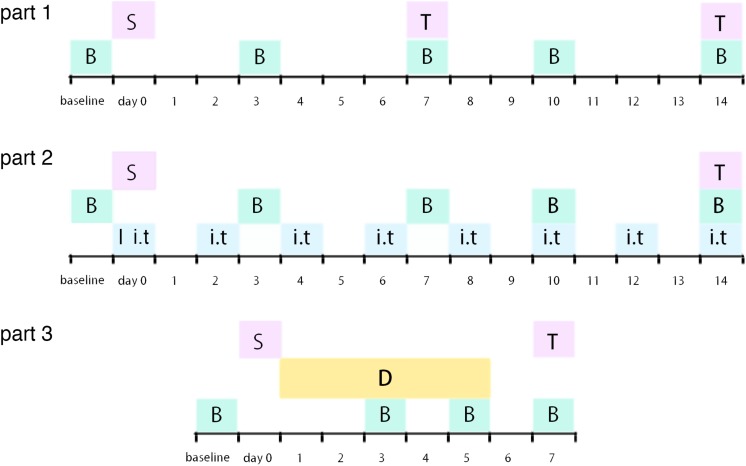
Illustration of experimental timeline S: surgery procedure; T: tissue collection; B: behavioral tests; I: catheter implantation; i.t.: intrathecal injection (RAP/3-MA/TM/4-PBA or vehicle); D: dexmedetomidine (or vehicle) administration.

Male Sprague–Dawley (SD) rats (180–230 g, Chang Sheng biological technology, China) were housed in a temperature- and humidity-controlled room with a 12-h light/dark cycle with chow and tap water available *ad libitum* in Benxi Medical and Pharmaceutical Research Base of Shengjing Hospital. Procedures of this study were in accordance with the recommendations in the guide for the care and use of laboratory animals of China Medical University. All surgical procedures were performed under 10% chloral hydrate and sevoflurane anesthesia, and all efforts were made to minimize animal’s suffering. The experimental protocols were approved by the Experimental Animal Committee of China Medical University (approved number: 2016PS013K). All rats were randomly assigned into different groups. Experimental timeline of this study was demonstrated as Figure 1.

### Spinal Nerve Ligation Model

According to the procedure originally proposed by Kim and Chung and described previously (Liu et al., 2017; Liu Y.D. et al., 2019), the left 5th lumbar spinal nerve was tightly ligated by a 4-0 silk suture and cut distal to the ligature under anesthesia. The surgical procedure for the sham group was identical to the SNL group, except that the spinal nerve was not ligated and cut.

### Intrathecal Drug Administration

Intrathecal injection was performed as described previously (Liu Y.D. et al., 2019). Briefly, laminectomy was performed at the L5 vertebra under anesthesia. PE-5 catheter was inserted into the subarachnoid space of the spinal cord at L4 level. The rapamycin (autophagy inducer, CST, 0.2 μg/10 μl), 3-MA (autophagy inhibitor, Selleck, 50 μM), 4-PBA (ER stress inhibitor, Selleck, 200 μM), and tunicamycin (ER stress inducer, Selleck, 25 μM) were intrathecally administered 10 μl on operation day and postoperative day 2, 4, 6, 8, 10, 12, and 14 in SNL + RAP, SNL + 3-MA, SNL + 4-PBA, and SNL + TM group, respectively. All the groups without specific noted harvested spinal cord (L3–L5) at postoperative 14 day. The sham + C group received sham operation and catheter implantation procedure. SNL + C group received spinal nerve ligation and catheter implantation. The sham + C and SNL + C groups received 10 μl saline via catheter on operation day and postoperative day 2, 4, 6, 8, 10, 12, and 14.

### Intraperitoneal Drug Administration

In this study, we intraperitoneally administered dexmedetomidine (30 μg/kg) from postoperative day 1 to day 5 in SNL + DEX group. sham + V and SNL + V groups received saline (3 ml/kg) intraperitoneally as vehicle from operation day to postoperative day 5. On operation day, dexmedetomidine or saline was intraperitoneally administered after 4 h later from surgery procedure.

### Behavior Tests

#### Mechanical Withdrawal Threshold (MWT)

To examine paw sensitivity in response to mechanical stimulus, MWT was applied as described previously (Liu Y.D. et al., 2019). MWT was considered as the minimum force (*g*) in which a von Frey filament (Stoelting Company, United States) triggers a positive paw withdrawal response via an up-and-down procedure. Each von Frey filament was held for approximate 5 s.

#### Thermal Withdrawal Latency

To examine paw sensitivity in response to thermal stimulus, the TWL assay was applied as described previously (Li and Zhang, 2018). The left hind paw of the animal was exposed to a heat stimulus via automatic plantar analgesia tester (BME-410C, China). The duration of paw withdrawal from the heat source was defined as TWL.

Before MWT and TWL tests on behavior test day, SD rats were placed in Plexiglas chambers, located on an elevated wire grid, and allowed to habituate for at least 1 h. The behavioral tests were carried out 2 days before surgery (baseline) and postoperative day 3, 7, 10, and 14. The cutoff value was 15 g for MWT and 30 s for TWL. For each trial, five mechanical/thermal stimuli at 5 min intervals were delivered. On behavior test day, if the rats need to receive drug/saline administration, the MWT and TWL tests were carried out 2 h before the drug/saline administration.

#### Western Blot

At the samples’ harvest time points, SD rats were sacrificed and the spinal cord lumbar segments (L3–L5) were carefully dissected and immediately frozen at −80°C until use. The specimens were homogenized in ice-cold RIPA lysis buffer (p0013B, Beyotime, China) in the presence of protease inhibitors (Solarbio, China) and incubated on ice for 30 min followed by centrifugation at 14,000 rpm for 45 min at 4°C. The supernatant fraction after centrifugation was taken for analysis. BCA protein assay kit (Solarbio, China) was used to determine the protein levels in supernatant. The samples were separated by 10% SDS-PAGE gel electrophoresis and were transferred to PVDF membranes (GE, United States). Bands were blocked with 5% BSA in TBST (0.1%Tween 20 in Tris-buffered saline) for 0.5 h at room temperature. Relative primary antibodies were incubated at 4°C overnight, respectively. Bands were washed with TBST and were incubated with HRP-conjugated second antibodies for 1 h at room temperature. Finally, these bands were detected by high-sig ECL Western blotting substrate (Tanon, China) with the chemiluminescence imaging systems (GE, United States; c300, Azure Biosystems, United States) and quantified with ImageJ software (NIH, United States).

The following primary antibodies and dilutions were used: rabbit Grp78 (1:2000, Abcam, United States), rabbit PERK (1:500, CST, United States), rabbit p-PERK (1:500, CST, United States), rabbit ATF4 (1:1000, Abcam, United States), rabbit ATF6 (1:1000, Abcam, United States), rabbit IRE1 (1:1000, Abcam, United States), rabbit p-IRE1 (1:1000, Abcam, United States), rabbit LC3 (1:2000, CST, United States), rabbit p62 (1:1000, CST, United States), rabbit FAM134B (1:1000, Abcam, United States), p-JNK (1:1000, CST, United States), cleaved caspas3 (1:1000, Abcam, United States), mouse GAPDH (1:8000, Solarbio, China), goat anti-rabbit IgG horseradish peroxidase, or goat anti-mouse IgG horseradish peroxidase (1:4000, Beyotime, China).

### Immunofluorescence Staining

Sprague–Dawley rats were deeply anesthetized and perfused transcardially with 0.9% NaCl solution, followed by cold 4% paraformaldehyde in 0.1 M PBS. Samples were removed, post-fixed in 4% paraformaldehyde fixative solution for 24 h, and cytoprotected with 30% sucrose in ddH2O for 24 h at 4°C. Samples of RVM were coronal sectioned on cryostat at 10-μm thickness. Coronal sections were incubated with primary antibody against Grp78 (ER stress marker, 1:100, Abcam, United States), followed by incubation with the FITC-conjugated secondary antibody (1:200, Proteintech, China). For double immunostaining, sections were sequentially incubated also with anti-NeuN (a neuronal marker, 1:100, MAB377, Millipore, United States) antibody followed by incubation with TRITC-conjugated secondary antibody (1:200, Proteintech, China). Cell nuclei were counterstained with DAPI (Beyotime, China) for 5 min.

### Statistical Analysis

The results show as the mean ± standard error of the mean (SEM). Analysis was performed using IBM SPSS Statistics 22 software. Western blot results were analyzed by one-way analysis of variance (ANOVA) following *post hoc* multiple comparison; MWT and TWL data were analyzed by two-way analysis of variance (ANOVA) following *post hoc* multiple comparison. *P* values < 0.05 were considered significant.

## Results

### SNL Induced Mechanical and Thermal Hypersensitivity

**FIGURE 2:**
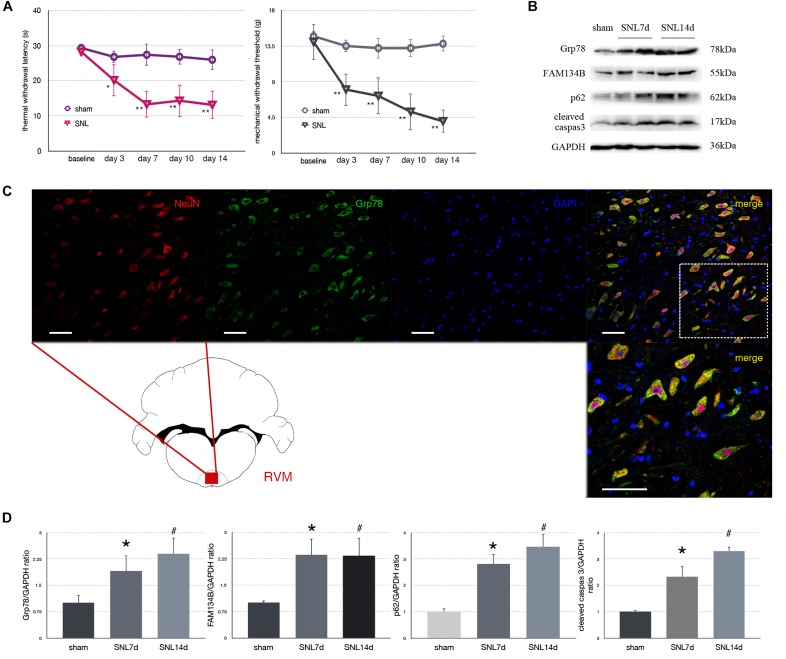
Effect of SNL on behavior tests, protein expression in spinal cord, and immunofluorescence staining in RVM (rostral ventromedial medulla). **(A)** TWL (thermal withdraw latency) and MWT (mechanical withdraw threshold) were used to evaluate the thermal and mechanical hyperalgesia in SNL rats. *p < 0.05 vs. sham group. **(B)** Western blot analysis of Grp78, FAM134B, p62, cleaved caspase-3 in the spinal cord of sham, SNL7d (SNL postoperative 7 days), and SNL14d group (SNL postoperative 14 days). GAPDH is used as an endogenous control. **(C)** Representative double immunofluorescence images of Grp78 (green) and NeuN (red) in RVM of SNL 14d are presented. Scale bar = 100 μm. **(D)** Western blot quantification of Grp78, FAM134B, p62, cleaved caspase-3 in the spinal cord of sham, SNL7day, SNL 14d groups. GAPDH is used as sample loading control. Data are presented as mean ± SD, *n* = 6 rats per group. *P < 0.05 SNL7d vs. sham group; ^#^*p* < 0.05 SNL14d vs. sham group. In behavior tests, **p < 0.01, ^##^*p* < 0.01.

To determine mechanical and thermal hypersensitivity following SNL, we measured MWT and TWL tests (Figure 2A). Our data showed that SNL induced significantly mechanical and thermal hypersensitivity (Figure 2A, ^∗^*p* < 0.05; ^∗∗^*p* < 0.01).

### SNL Induced ER Stress and Autophagy Impairment in Spinal Cord

Spinal nerve ligation induced Grp78 (sham vs. SNL7d, *p* = 0.006; sham vs. SNL14d, *p* = 0.001; *F* = 9.338) high expression in L3–L5 spinal cord (Figures 2B,D), suggesting that ER stress participates in nociceptive ascending modulation system. Expression of FAM134B (sham vs. SNL7d, *p* = 0.005; sham vs. SNL14d, *p* = 0.001; *F* = 11.284) was significantly upregulated in spinal cord (Figures 2B,D), suggesting that the level of ER sheets that bind to autophagosomes was increased. Our data showed that p62 (sham vs. SNL7d, *p* = 0.016; sham vs. SNL14d, *p* = 0.001; *F* = 11.336) (Figures 2B,D) was increased following SNL compared to sham group, suggesting that autophagy was impaired. Cleaved caspase-3 (sham vs. SNL7d, *p* = 0.017; sham vs. SNL14d, *p* < 0.001; *F* = 11.346) was increased compared to sham group in spinal cord, indicating that apoptosis level was elevated in SNL rats compared to the sham group. In addition, Grp78 was expressed in spinal cord and RVM neurons (Figure 2C), indicating that ER stress participates in nociceptive descending modulation system in SNL rats.

### Effect of 4-PBA Injection on Pain-Related Tests and Protein Expressions in SNL Rats

**FIGURE 3:**
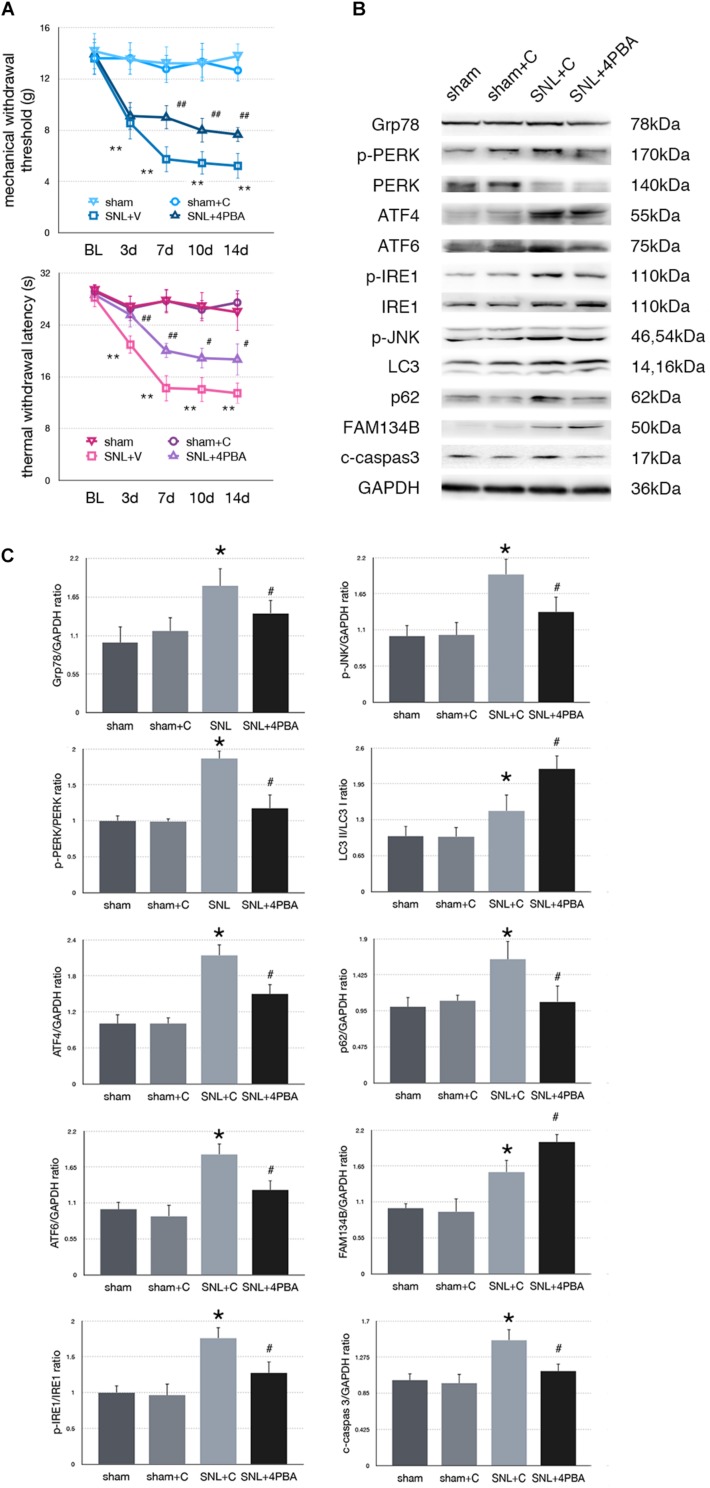
Effect of 4-PBA on pain-related behavior tests and protein expression. **(A)** TWL and MWT in sham, sham + C, SNL + C, and SNL + 4-PBA. **(B)** Representative immunoblots of related protein expression are shown. **(C)** Quantifications of Western blot in sham, sham + C, SNL + C, and SNL + 4-PBA groups. 4-PBA leads to alleviate ER stress and induces ER-phagy increase in spinal cord. Data are presented as mean ± SD, *n* = 6 rats per group. sham vs. sham + C have no significant difference; *p < 0.05 SNL + C group vs. sham + C group; #*p* < 0.05 SNL + 4-PBA vs. SNL + C group. In behavior tests, **p < 0.01, ^##^*p* < 0.01.

To determine whether alleviated ER stress could modulate ER-phagy, we intrathecally injected 4-PBA (4-phenylbutyric acid, an ER stress inhibitor) to ameliorate ER stress in SNL rats. Significant analgesia effects evaluated by MWT and TWL tests were observed at post-operation time points (Figure 3A). There was no significant difference between sham and sham + C group (*p* > 0.05). Administration of 4-PBA led to Grp78 (sham + C vs. SNL + C, *p* = 0.013; SNL + C vs. SNL + 4PBA, *p* = 0.036; *F* = 4.632) and UPR sensors (sham + C, SNL + C, SNL + 4PBA, ANOVA, p-PERK/PERK, *p* = 0.036, *F* = 4.632; ATF4, *p* = 0.009, *F* = 8.026; ATF6, *p* = 0.006, *F* = 9.003; p-IRE/IRE, *p* = 0.001, *F* = 14.055; p-JNK, *p* = 0.022, *F* = 5.696) decreasing compared to the SNL + C group. Moreover, application of 4-PBA induced FAM134B (sham + C vs. SNL + C, *p* = 0.005; SNL + C vs. SNL + 4PBA, *p* < 0.001; *F* = 14.058) and LC3-II/LC3-I (sham + C vs. SNL + C, *p* = 0.013; SNL + C vs. SNL + 4PBA, *p* = 0.021; *F* = 16.761) increased and p62 (sham + C vs. SNL + C, *p* = 0.013; SNL + C vs. SNL + 4PBA, *p* = 0.012; *F* = 5.654) decreased compared to SNL + C group, indicating that ER-phagy level elevated following ameliorated ER stress in SNL rats. Cleaved caspase-3 (sham + C vs. SNL + C, *p* = 0.008; SNL + C vs. SNL + 4PBA, *p* = 0.035; *F* = 5.221) was decreased in the SNL + 4PBA group compared to the SNL + C group, suggesting that alleviated ER stress led to the decrease in apoptosis (Figures 3B,C).

### Effect of Tunicamycin Injection on Pain-Related Tests and Protein Expressions in SNL Rats

**FIGURE 4:**
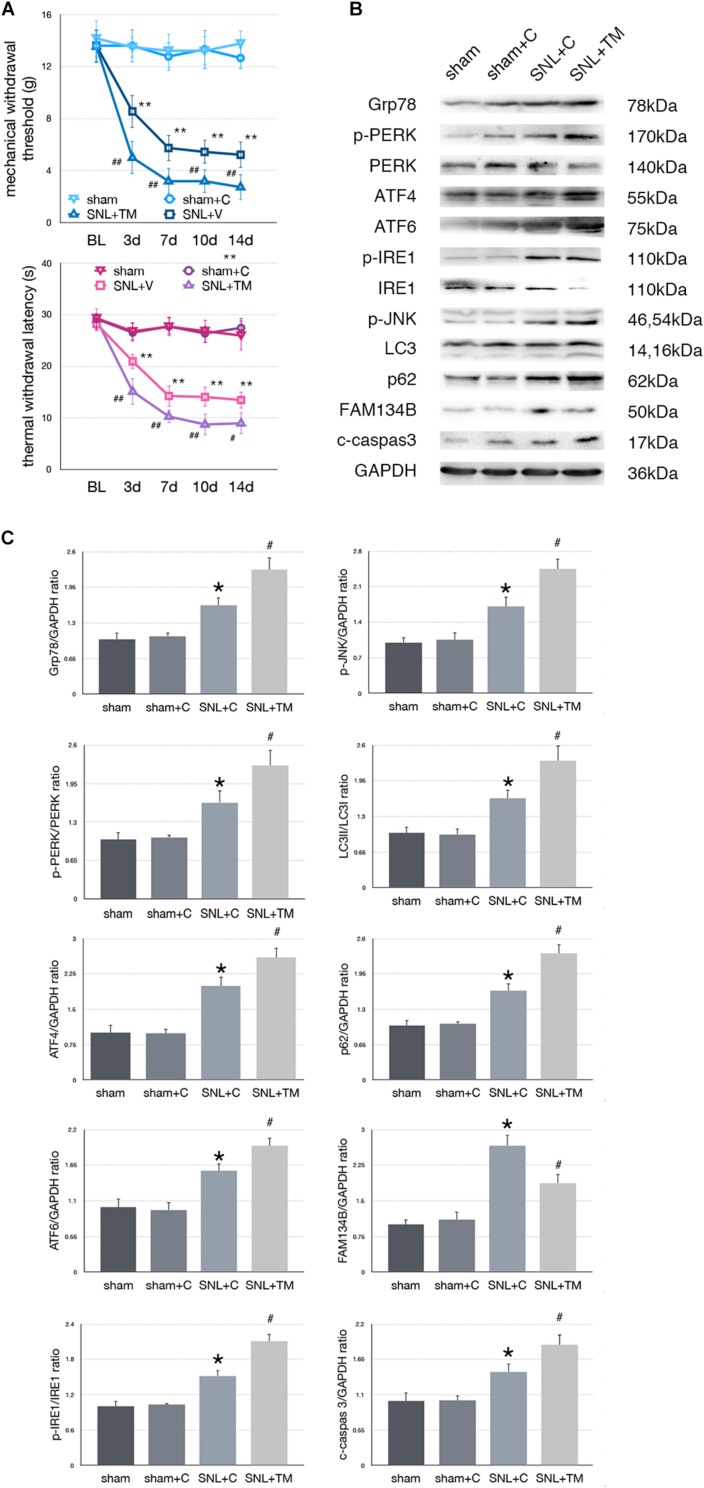
Effect of tunicamycin on pain-related behavior tests and protein expression. **(A)** Tunicamycin deteriorates TWL and MWT tests. **(B)** Western blot analysis of protein expressions in spinal cord were shown. **(C)** Quantifications of Western blot in sham, sham + C, SNL + C, and SNL + TM groups. Application of tunicamycin alters protein expressions in spinal cord are shown. Data are presented as mean ± SD, *n* = 6 rats per group. sham vs. sham + C have no significant difference; *p < 0.05 SNL + C group vs. sham + C group; ^#^ *p* < 0.05 SNL + TM vs. SNL + C group. In behavior tests, **p < 0.01, ^##^*p* < 0.01.

To determine whether excessive ER stress impacted ER-phagy, we intrathecally injected tunicamycin (TM, an ER stress inducer) to further raise ER stress level following SNL. There were no significant differences between sham and sham + C group in MWT and TWL tests (*p* > 0.05). Compared with the SNL + C group, SNL + TM exacerbated pain related behavior tests (Figure 4A, ^∗^*p* < 0.05; ^∗∗^*p* < 0.01), increased ER stress (Grp78, sham + C vs. SNL + C, *p* = 0.025; SNL + C vs. SNL + TM, *p* = 0.011; *F* = 17.282) and UPR pathway level (Figure 4B, sham + C, SNL + C, SNL + TM, ANOVA, p-PERK/PERK, *p* = 0.001, *F* = 17.965; ATF4, *p* = 0.001, *F* = 21.815; ATF6, *p* = 0.001, *F* = 17.955; p-IRE/IRE, *p* = 0.001, *F* = 10.046; p-JNK, *p* < 0.001, *F* = 14.688). Moreover, our data demonstrated that the expression level of FAM134B (sham + C vs. SNL + C, *p* < 0.001; SNL + C vs. SNL + TM, *p* = 0.004; *F* = 20.917) was significantly decreased, and LC3-II/LC3-I (sham + C vs. SNL + C, *p* = 0.04; SNL + C vs. SNL + TM, *p* = 0.034; *F* = 11.172) and p62 (sham + C vs. SNL + C, *p* = 0.019; SNL + C vs. SNL + TM, *p* = 0.011; *F* = 18.107) were increased in the SNL + TM group, indicating that ER-phagy level was decreased following tunicamycin injection in SNL rats. Compared with the SNL + C group, cleaved caspase-3 (sham + C vs. SNL + C, *p* = 0.018; SNL + C vs. SNL + TM, *p* = 0.024; *F* = 15.406) was significantly increased in the SNL + TM group (Figures 4B,C).

### Effect of Rapamycin Injection on Nociceptive Behavior Tests and Protein Expressions in SNL Rats

**FIGURE 5:**
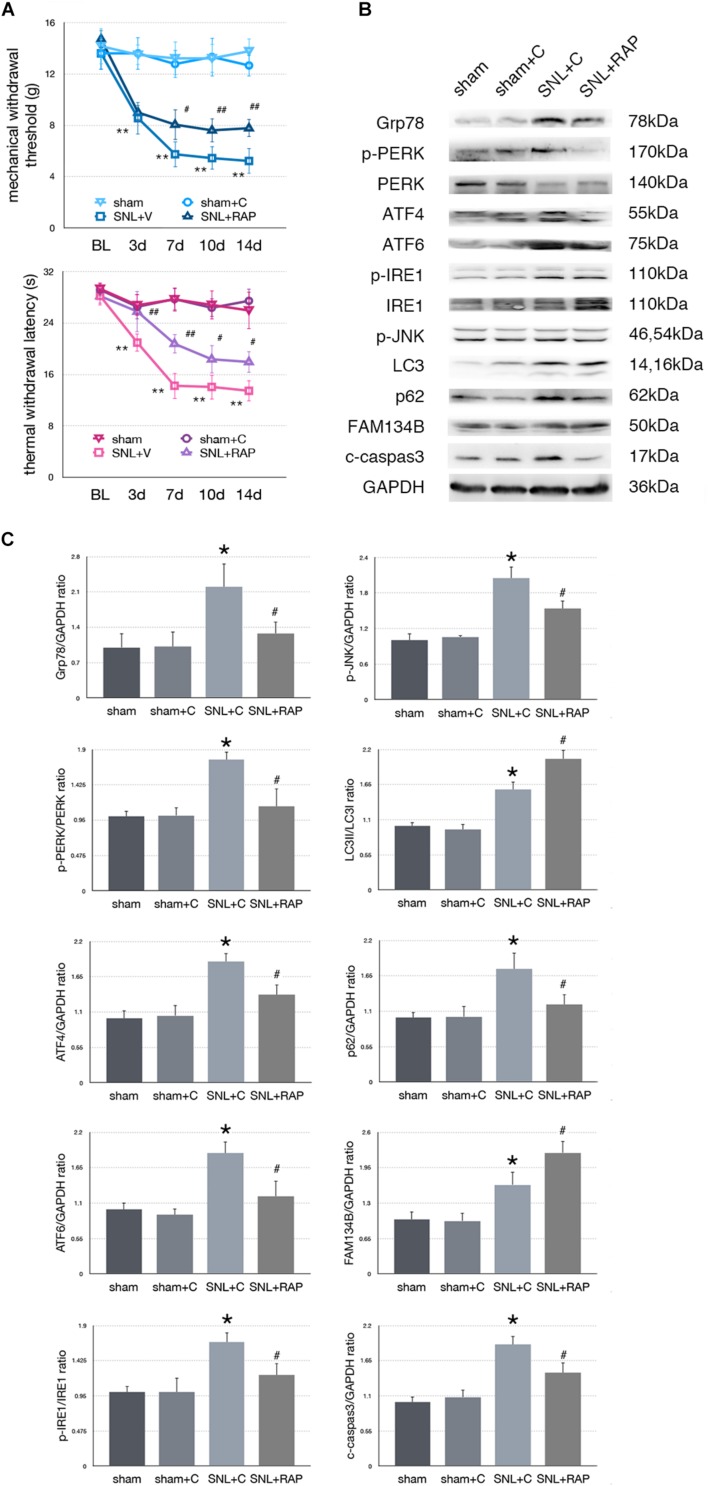
Effect of rapamycin on pain-related behavior tests and protein expression. **(A)** Application of rapamycin induces pain relief effect via TWL and MWT tests. **(B)** Rapamycin counteracts SNL-induced ER stress and increases ER-phagy in spinal cord. **(C)** Quantifications of Western blot in sham, sham + C, SNL + C, and SNL + RAP groups. Protein expressions in spinal cord are shown. Data are presented as mean ± SD, *n* = 6 rats per group. sham vs. sham + C have no significant difference; *p < 0.05 SNL + C group vs. sham + C group; ^#^*p* < 0.05 SNL + 4-PBA vs. SNL + C group. In behavior tests, **p < 0.01, ^##^*p* < 0.01.

To determine whether autophagy counteracted SNL-induced ER stress, we intrathecally administered rapamycin (RAP, an autophagy inducer) to induce autophagy in SNL rats. Rapamycin alleviated hypersensitivity induced by SNL. As shown in Figure 5A, MWT and TWL were significantly increased (^∗^*p* < 0.05; ^∗∗^*p* < 0.01) in SNL + RAP compared with the SNL + C group. Rapamycin administration increased FAM134B (sham + C vs. SNL + C, *p* = 0.024; SNL + C vs. SNL + RAP, *p* = 0.040; *F* = 12.348) and LC3-II/LC3-I (sham + C vs. SNL + C, *p* = 0.005; SNL + C vs. SNL + RAP, *p* = 0.019; *F* = 20.607), decreased p62 (sham + C vs. SNL + C, *p* = 0.004; SNL + C vs. SNL + RAP, *p* = 0.019; *F* = 7.103) expression ER-phagy and autophagy level, and decreased ER stress (Grp78, sham + C vs. SNL + C, *p* = 0.007; SNL + C vs. SNL + TM, *p* = 0.023; *F* = 5.949) and UPR (sham + C, SNL + C, SNL + TM, ANOVA, p-PERK/PERK, *p* = 0.002, *F* = 13.337; ATF4, *p* = 0.002, *F* = 12.128; ATF6, *p* = 0.002, *F* = 11.996; p-IRE/IRE, *p* = 0.009, *F* = 7.919; p-JNK, *p* = 0.007, *F* = 8.730) sensor levels in spinal cord. The expression level of cleaved caspase-3 (sham + C vs. SNL + C, *p* = 0.004; SNL + C vs. SNL + RAP, *p* = 0.005; *F* = 9.306) was decreased in the SNL + RAP group compared with the SNL + C group (Figures 5B,C).

### Effect of 3-MA Injection on Pain-Related Tests and Protein Expressions in SNL Rats

**FIGURE 6:**
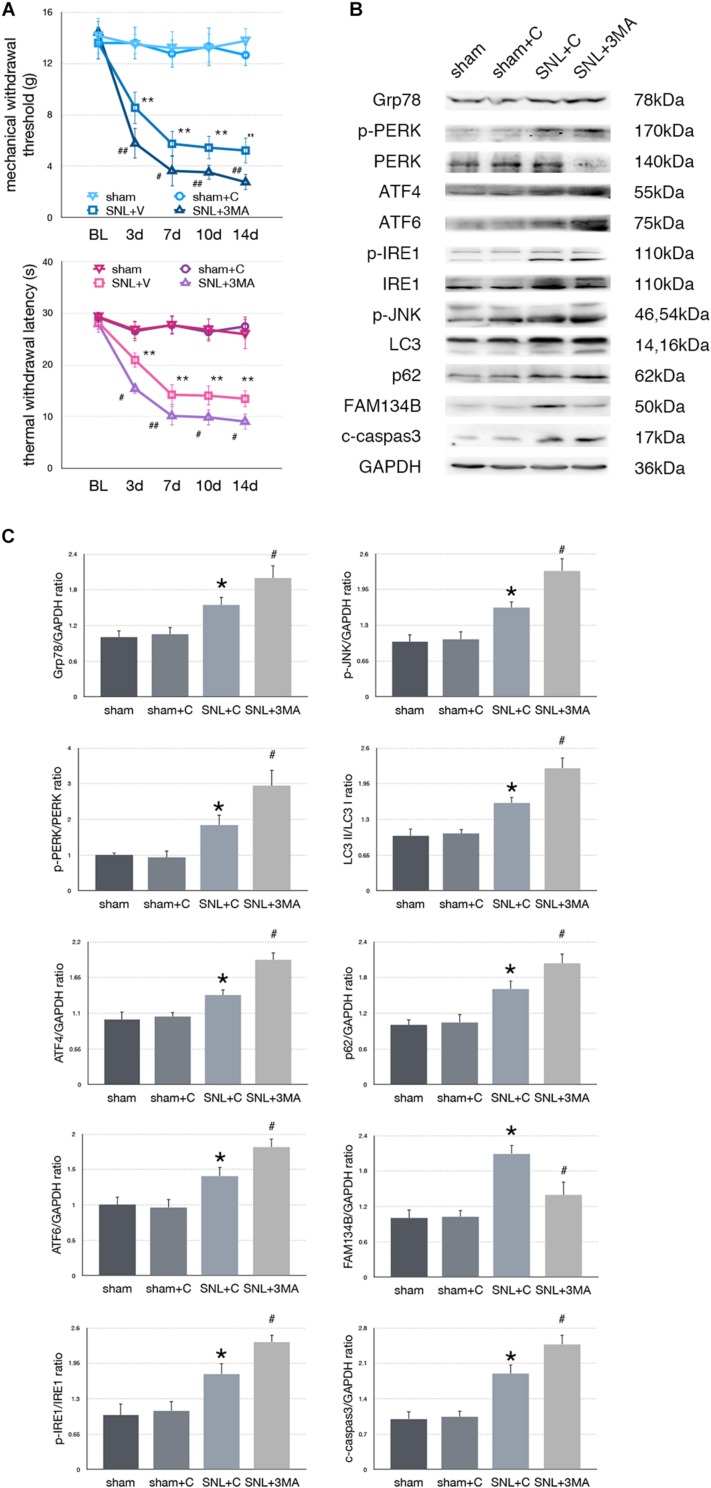
Effect of 3-MA on pain related behavior tests and protein expression. **(A)** 3-MA injection deteriorates TWL and MWT tests in SNL rats. **(B)** 3-MA induces pro-death effect via exacerbating ER stress and ER-phagy impairment induced by SNL in spinal cord. **(C)** Quantifications of Western blot in sham, sham + C, SNL + C, and SNL + 3-MA groups. Protein expressions in spinal cord are shown. Data are presented as mean ± SD, *n* = 6 rats per group. sham vs. sham + C have no significant difference; *p < 0.05 SNL + C group vs. sham + C group; ^#^*p* < 0.05 SNL + 3-MA vs. SNL + C group. In behavior tests, **p < 0.01, ^##^*p* < 0.01.

To determine whether aggravated autophagy impairment could modulate ER stress, SNL rats were intrathecally injected with 3-MA (3-Methyladenine, an autophagy inhibitor) to block autophagy in the SNL + 3-MA group. Application of 3-MA deteriorated MWT and TWL tests (Figure 6A, ^∗^*p* < 0.05; ^∗∗^*p* < 0.01). Intrathecal administration of 3-MA significantly increased expressions of Grp78 (sham + C vs. SNL + C, *p* = 0.007; SNL + C vs. SNL + 3-MA, *p* = 0.015; *F* = 12.437), p-PERK/PERK (sham + C vs. SNL + C, *p* = 0.044; SNL + C vs. SNL + 3-MA, *p* = 0.021; *F* = 22.028), ATF4 (sham + C vs. SNL + C, *p* = 0.029; SNL + C vs. SNL + 3-MA, *p* = 0.002; *F* = 13.835), ATF6 (sham + C vs. SNL + C, *p* = 0.031; SNL + C vs. SNL + 3-MA, *p* = 0.043; *F* = 11.040), p-IRE1/IRE1 (sham + C vs. SNL + C, *p* = 0.011; SNL + C vs. SNL + 3-MA, *p* = 0.022; *F* = 18.656), p-JNK (sham + C vs. SNL + C, *p* = 0.040; SNL + C vs. SNL + 3-MA, *p* = 0.028; *F* = 12.133), LC3-II/LC3-I (sham + C vs. SNL + C, *p* = 0.014; SNL + C vs. SNL + 3-MA, *p* = 0.008; *F* = 20.708), p62 (sham + C vs. SNL + C, *p* = 0.024; SNL + C vs. SNL + 3-MA, *p* = 0.040; *F* = 12.769), and cleaved caspase-3 (sham + C vs. SNL + C, *p* = 0.007; SNL + C vs. SNL + 3-MA, *p* = 0.039; *F* = 18.168) and decreased expressions of FAM134B (sham + C vs. SNL + C, *p* = 0.001; SNL + C vs. SNL + 3-MA, *p* = 0.013; *F* = 10.746) compared with SNL + C group (Figures 6B,C). Our data showed that 3-MA enhanced the level of ER stress and decreased ER-phagy in spinal cord.

### Dexmedetomidine Relieved SNL-Induced Neuropathic Pain-Related Behavior Tests

**FIGURE 7:**
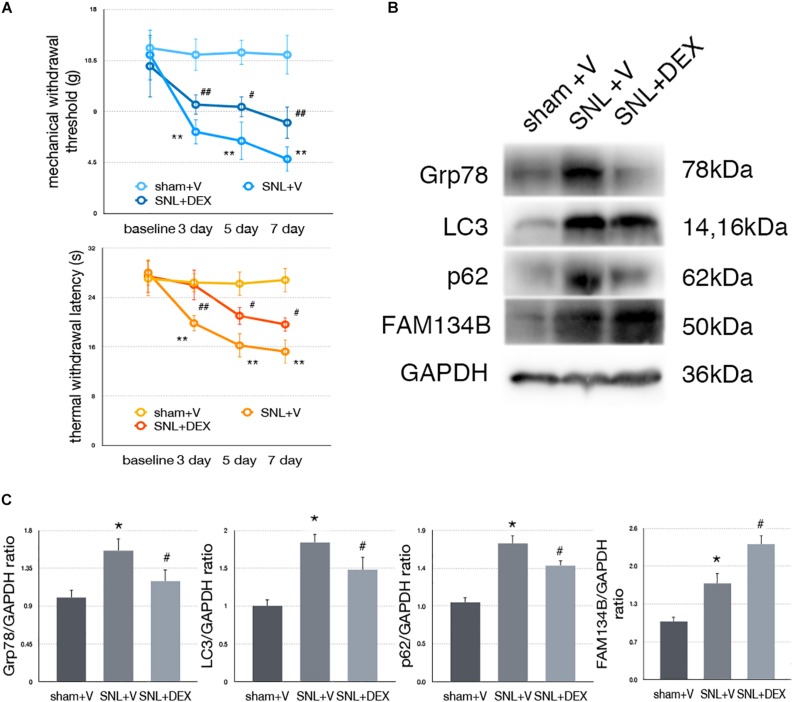
Dexmedetomidine enhanced ER-phagy and alleviated ER stress to provide analgesic effect. **(A)** Dexmedetomidine improved MWT and TWL tests. **(B)** Protein expressions of Grp78, LC3, p62, and FAM134B in sham + V, SNL + V, and SNL + DEX group. **(C)** Quantifications of Western blot in sham + V, SNL + V, and SNL + DEX group. Data are presented as mean ± SD, *n* = 6 rats per group. *p < 0.05 SNL + V group vs. sham + V group; ^#^*p* < 0.05 SNL + V vs. SNL + DEX group. In behavior tests, **p < 0.01, ^##^*p* < 0.01.

We confirm the analgesic effect of dexmedetomidine in SNL rats. Our data demonstrated that administration of dexmedetomidine led to improved MWT and TWL tests (Figure 7A, ^∗^*p* < 0.05; ^∗∗^*p* < 0.01).

### Dexmedetomidine Provided Analgesic Effect Through Alleviating ER Stress to Induce ER-phagy in SNL Rats

To determine whether dexmedetomidine alleviates neuropathic pain via ER stress and ER-phagy, we carried out Western blot analysis to evaluate the expression of Grp78 (sham + V vs. SNL + V, *p* = 0.009; SNL + V vs. SNL + DEX, *p* = 0.045; *F* = 7.376), LC3-I (sham + V vs. SNL + V, *p* = 0.001; SNL + V vs. SNL + DEX, *p* = 0.027; *F* = 19.043), p62 (sham + V vs. SNL + V, *p* = 0.004; SNL + V vs. SNL + DEX, *p* = 0.025; *F* = 10.997), and FAM134B (sham + V vs. SNL + V, *p* = 0.012; SNL + V vs. SNL + DEX, *p* = 0.010; *F* = 26.361). Our data suggested that dexmedetomidine induced ER-phagy to relieve ER stress in the spinal cord of SNL rats (Figures 7B,C). The raw data of this study was available as Supplementary Material.

## Discussion

Cellular stress or cytotoxic proteins lead to prominent ER stress (Zhou et al., 2017). UPR is regarded as a part of ER stress. A high level or aggressively prolonged UPR signaling is closely related to apoptosis, however, a moderate or short-term UPR signaling results in restoring cell homeostasis and survival (Senft and Ronai, 2015; Zhang et al., 2015; Colla, 2019; Srikanth et al., 2019). Studies demonstrated that ER stress and UPR might be involved in the induction and maintenance of neuropathic pain (Liu Y.D. et al., 2019). Moreover, a disturbance in UPR pathway might render neurons vulnerable to neuropathic pain stimuli (Huang et al., 2015; Zhou et al., 2017; Kang et al., 2018).

The link between autophagy and ER stress via UPR has been investigated substantially (Deegan et al., 2013; Huang et al., 2013; Lupachyk et al., 2013; Sisinni et al., 2019; Yang et al., 2019). PERK pathway is essential for autophagy induction following ER stress (Lee et al., 2019). Furthermore, ATF4 and its downstream factor C/EBP homologous protein (CHOP) were capable of modulating ATG (autophagy related) genes. Also, the IRE1 pathway is implicated in the autophagy induction process (Chen et al., 2019).

Autophagy provides a homeostatic mechanism in response to ER stress and UPR. Autophagy could be non-selective or selective (Chino et al., 2019; Forrester et al., 2019; Geronimo-Olvera and Massieu, 2019; Wilkinson, 2019a; Yang et al., 2019). In this study, we examined the expression level of FAM134B to evaluate the ER-phagy. FAM134B is an ER-resident receptor that facilitates ER degradation by autophagy via binding to autophagosomes (Murrow and Debnath, 2013; Khaminets et al., 2015; Grumati et al., 2018; Liang et al., 2018; Ding et al., 2019; Forrester et al., 2019; Wilkinson, 2019a, b). Our data showed that Grp78, FAM134B, p62, and cleaved caspase-3 were increased in the spinal cord of the SNL group compared with the sham group at postoperative days 7 and 14. Our data showed for the first time that ER-phagy was modulated in the spinal cord of SNL rats. It suggested that the altered ER-phagy process was related to SNL-induced ER stress. However, ER-phagy degradation impairment in SNL-induced neuropathic pain led to apoptosis increase compared with the sham group.

Nociceptive descending system provides integration message from thalamus, cerebral cortex, and other important regions via rostral ventromedial medulla (RVM) (Chen et al., 2018), which is a critical region for the neuropathic pain nociceptive descending modulation system, before it reaches the spinal cord (Chen et al., 2018). Whether ER stress participates in the descending nociceptive sensory information system is determined. Immunofluorescence tests were carried out to investigate whether ER stress was altered in neurons. Our data demonstrated that Grp78 was mainly expressed in RVM neurons. Studies demonstrated that XBP-1 (spliced x-box binding protein 1, an IRE-1 downstream protein), p-eIF2α, ATF6, and Grp78 double immunofluorescence stained with NeuN in the spinal cord of SNL rats, respectively (Inceoglu et al., 2015; Zhang et al., 2015; Ge et al., 2018). Our previous study demonstrated that LC3 was mainly expressed in neuronal cell. These results indicated that ER stress and the autophagy process might be a promising treatment target in neuronal cells of the neuropathic pain animal model.

To determine the relationship between ER stress and ER-phagy, ER stress inducer and inhibitor were intrathecally administered, respectively. Application of 4-PBA (ER stress inhibitor) significantly alleviated mechanical and thermal hypersensitivity. Compared with SNL + C, the expressions of Grp78, p-PERK/PERK, ATF4, ATF6, p-IRE1/IRE1, p-JNK, p62, and cleaved caspase-3 were significantly decreased and FAM134B and LC3-II/LC3-I were significantly increased in the SNL + 4-PBA group. These results demonstrated that alleviated ER stress level provided a pro-survival effect via inactivation of UPR and inhibition of ER stress to enhance ER-phagy in spinal cord of SNL rats. On the other hand, administration of tunicamycin (ER stress inducer) significantly deteriorated mechanical and thermal hypersensitivity. Tunicamycin induced full-scale activation of UPR and ER stress in SNL rats. Furthermore, the results of LC3, p62, FAM134B, and cleaved caspase-3 suggested that intrathecal injection with tunicamycin rendered deterioration of ER-phagy impairment and provided a pro-death process in SNL-induced neuropathic pain.

To determine whether altered autophagy modulates the level of ER stress in SNL-induced neuropathic pain as a feedback mechanism, intrathecal administration of rapamycin and 3-MA was applied, respectively. Administration of rapamycin increased the expressions of LC3-II/LC3-I and FAM134B and decreased the level of p62, indicating that rapamycin increased the ER-phagy level in SNL rats. Furthermore, intrathecal injection with rapamycin decreased ER stress and inhibited UPR pathways in the spinal cord of SNL rats. On the contrary, 3-MA administration worsened mechanical and thermal hypersensitivity and autophagy impairment in SNL rats. Blocking the autophagy process induced ER stress, and UPR and apoptosis increased, suggesting a negative feedback mechanism between ER stress and ER-phagy. Without efficient autophagy, degrading aggregate cytotoxic proteins resulted in enhancing the level of ER stress as a feedback and led to apoptosis in SNL-induced neuropathic pain.

In this study, our data demonstrated that pain-related behavior tests were improved after receiving rapamycin/4-PBA injection individually. The outcomes were satisfying in the SNL animal model. However, pain physicians are concerned about clinical outcomes of pharmacotherapies targeting autophagy or ER stress. For instance, rapamycin ester derivative easily crosses the blood–brain barrier and acts on both the peripheral and central nervous system. Studies showed that rapamycin administration received clinical benefits in neuropathic pain treatment (Gallagher et al., 2016). On the other hand, pharmacotherapy such as *N*-acetyl-L-cysteine (NAC) provides an ER stress alleviation effect that might be clinically beneficial with regard to neuropathic pain (ClinicalTrials.gov Identifier: NCT01840345). Anyway, there is no denying the fact that to meet the goal of individualized and multidisciplinary medicine for neuropathic pain, patients still need more options and adjuvants in clinic (Chakravarthy et al., 2018).

Dexmedetomidine is a highly effective and highly selective α2 adrenergic receptor agonist (Yang et al., 2018). Dexmedetomidine, a sedative drug with analgesic effect, is widely used in the operation room and intensive care unit. Furthermore, studies showed that dexmedetomidine might be a promising pharmacotherapy and adjuvant for neuropathic pain (Huang et al., 2017; Yang et al., 2018). However, the underlying mechanism needs further research. Our data demonstrated that Grp78, p62, and LC3-1 were decreased and FAM134B was increased in the SNL + DEX group compared with the SNL + V group, suggesting that dexmedetomidine induced ER-phagy to alleviate ER stress level in the spinal cord of SNL rats.

In all, SNL induced ER stress, ER-phagy impairment, and apoptosis in spinal cord. ER-phagy could provide a pro-survival degradation mechanism in response to ER stress in SNL rats. Furthermore, our data suggested that ER-phagy impairment was both a trigger and an effector of ER stress via UPR pathways in SNL rats. In addition, ER stress is expressed in RVM and spinal cord neurons in SNL rats, suggesting that ER stress was altered in the descending nociceptive system. Above all, we hypothesize that ER-phagy might play an important role in this co-regulation condition and provide a novel treatment target. In addition, dexmedetomidine targets ER-phagy to offer a novel insight into understanding its pharmacological mechanism as a pharmacotherapy of neuropathic pain.

## Data Availability Statement

The raw data supporting the conclusions of this article will be made available by the authors, without undue reservation, to any qualified researcher.

## Ethics Statement

The animal study was reviewed and approved by Experimental Animal Committee of China Medical University (approved number:2016PS013K).

## Author Contributions

YL carried out the animal experiments, Western blot, immunofluorescence staining, and study design. SW helped analyze data, study design, and start manuscript. ZW helped carry out behavior tests and data analysis. MD helped carry out and confirm Western blot picture. XL helped carry out immunofluorescence staining, animal tests, and data analysis. JG helped carry out immunofluorescence staining and animal behavior tests. GH helped Western blot procedure and study design. PZ designed and supported this study.

## Conflict of Interest

The authors declare that the research was conducted in the absence of any commercial or financial relationships that could be construed as a potential conflict of interest.
