# Supplementary material for: Dexmedetomidine Alleviated Endoplasmic Reticulum Stress via Inducing ER-phagy in the Spinal Cord of Neuropathic Pain Model
Source: Front Neurosci. 2020 Feb 28;14:90. doi: 10.3389/fnins.2020.00090 (PMC7058658; doi:10.3389/fnins.2020.00090)

Supplementary Material

Raw data of Western Blot images

Figure 1

Grp78

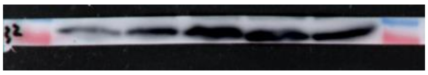

Fam134b

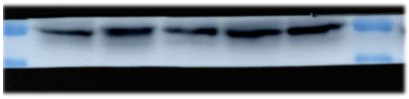

p62

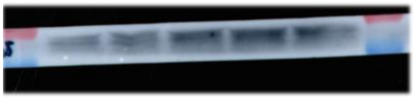

Cleaved-caspas3

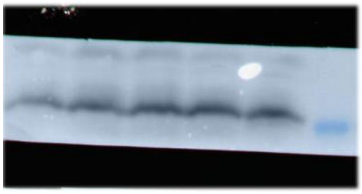

gapdh

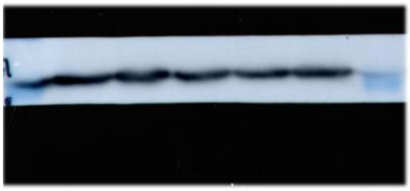

Figure 2

Grp78

p-perk

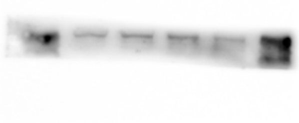

perk

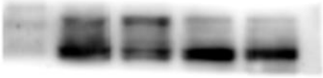

atf4

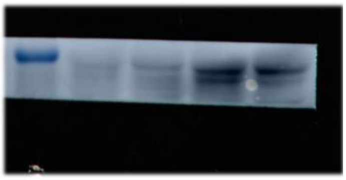

atf6

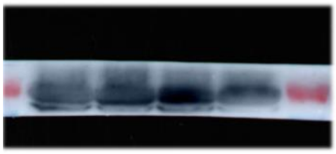

p-ire

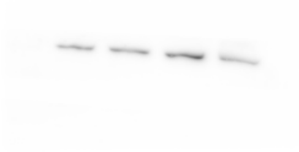

ire

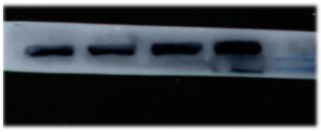

p-jnk

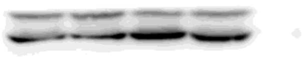

lc3

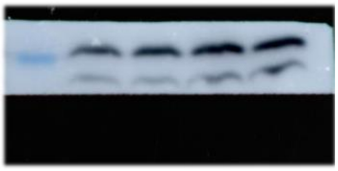

p62

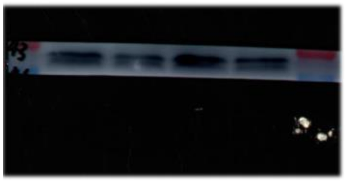

fam134b

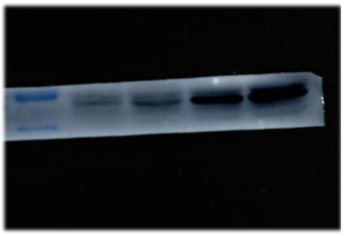

c-caspas3

gapdh

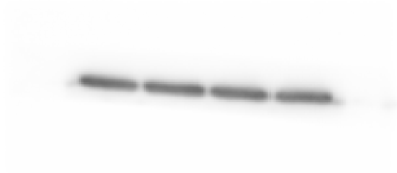

Figure 3

Grp78

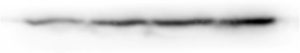

p-perk

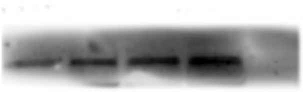

perk

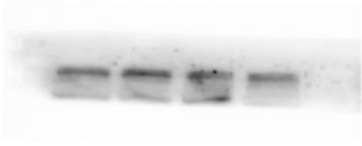

atf4

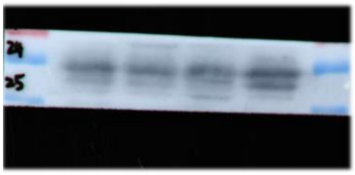

atf6

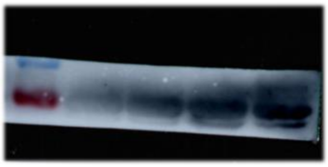

p-ire

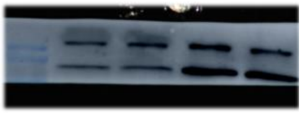

ire

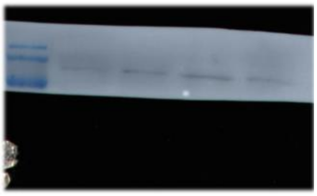

p-jnk

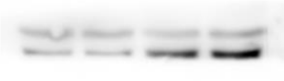

lc3

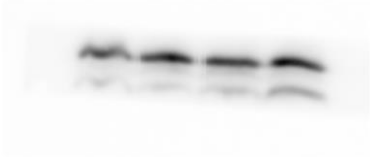

p62

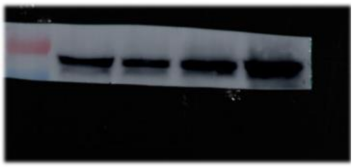

fam134b

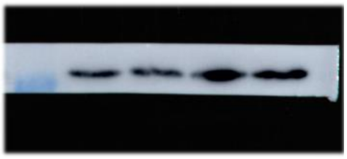

c-caspas3

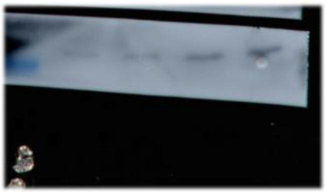

gapdh

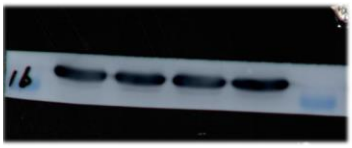

Figure 4

Grp78

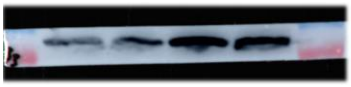

p-perk

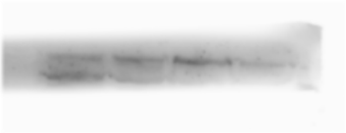

perk

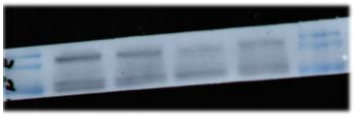

atf4

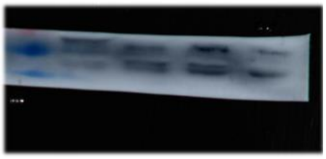

atf6

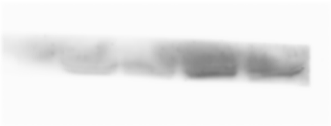

p-ire

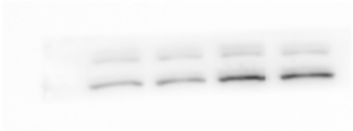

ire

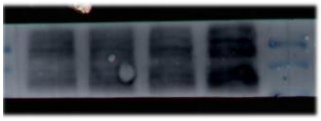

p-jnk

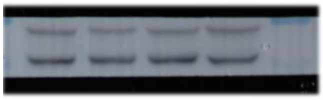

lc3

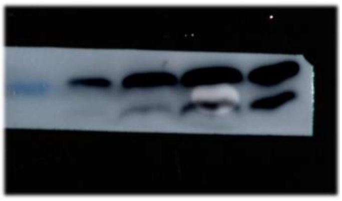

p62

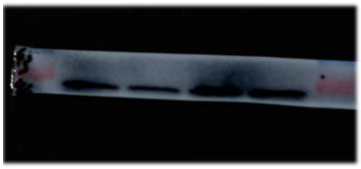

fam134b

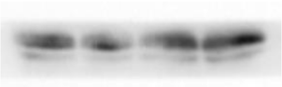

c-caspas3

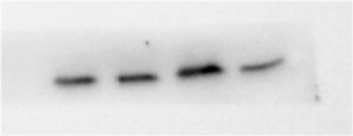

gapdh

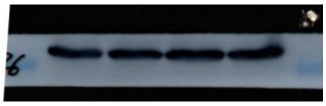

Figure 5

Grp78

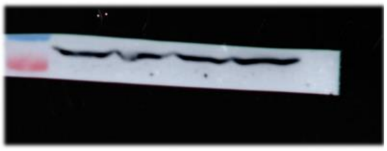

p-perk

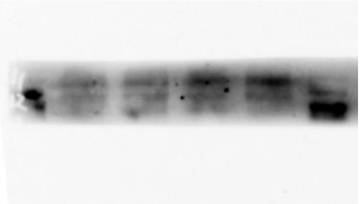

perk

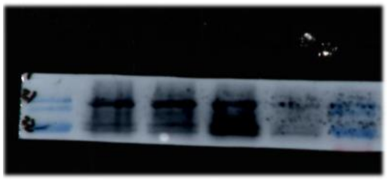

atf4

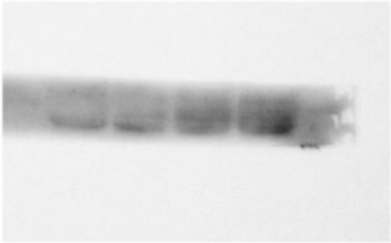

atf6

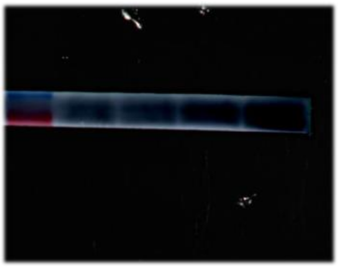

p-ire

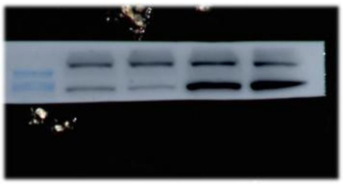

ire

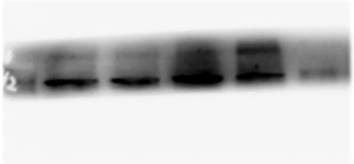

p-jnk

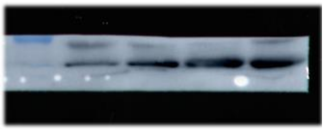

lc3

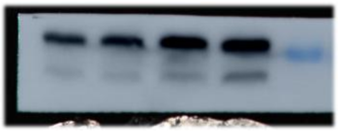

p62

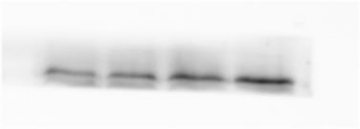

fam134b

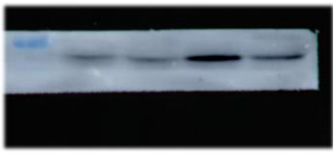

c-caspas3

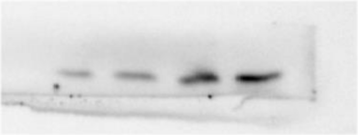

gapdh

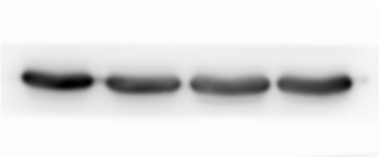

Figure 6

Grp78

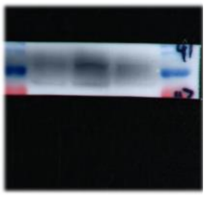

Lc3

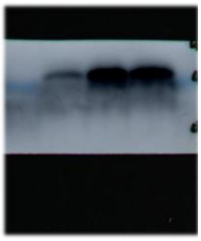

P62

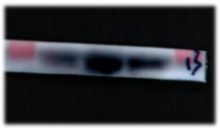

Fam134b

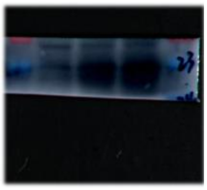

gapdh

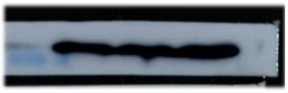

Supplement: Supplementary file 1 [file Data_Sheet_1.pdf]
